# Porcine reproductive and respiratory syndrome virus activates the pentose phosphate pathway via the ROS/HIF-1α/G6PD axis to promote viral replication

**DOI:** 10.1080/21505594.2025.2585639

**Published:** 2025-11-07

**Authors:** Ying-Xian Ma, Jia-Ming Yang, Hang-Tian Mei, Lei Zeng, Guo-Yu Yang, Jiang Wang, Sheng-Li Ming, Bei-Bei Chu

**Affiliations:** a College of Veterinary Medicine, Henan Agricultural University, Zhengzhou, Henan, China; b Key Laboratory of Animal Biochemistry and Nutrition, Ministry of Agriculture and Rural Affairs, Zhengzhou, Henan, China; c Key Laboratory of Veterinary Biotechnology of Henan Province, Henan Agricultural University, Zhengzhou, Henan, China; d State Key Laboratory of Membrane Biology, School of Pharmaceutical Sciences, Tsinghua University, Beijing, China; e Key Laboratory for Animal Pathogens and Biosafety, Ministry of Education, Zhengzhou, Henan, China; f Longhu Advanced Immunization Laboratory, Henan agricultural University, Zhengzhou, Henan, China; g International Joint Research Center of National Animal Immunology, Henan Agricultural University, Zhengzhou, Henan, China

**Keywords:** PRRSV, PPP, G6PD, HIF-1α, ROS

## Abstract

Porcine reproductive and respiratory syndrome virus (PRRSV), a highly contagious pathogen in swine, poses significant economic challenges to global pork production. This study elucidated the regulatory interplay between PRRSV infection and the pentose phosphate pathway (PPP), a critical metabolic axis for anabolism. Comparative metabolomic profiling of porcine alveolar macrophages (PAMs) pre- and post-PRRSV infection demonstrated marked upregulation of PPP activity, concomitant with elevated levels of nucleotide biosynthesis. This metabolic shift was driven by PRRSV-induced upregulation of glucose-6-phosphate dehydrogenase (G6PD), the PPP’s rate-limiting enzyme. Mechanistic investigations revealed that PRRSV infection stimulated hypoxia-inducible factor 1α (HIF-1α) expression, which transcriptionally activates G6PD. Genetic silencing of HIF-1α abolished PRRSV-mediated G6PD induction. Furthermore, reactive oxygen species (ROS) accumulation was identified as the upstream regulator of HIF-1α activation during PRRSV infection. Pharmacological ROS scavenging disrupted the ROS/HIF-1α/G6PD signaling cascade, diminished NADPH and reduced glutathione production, and consequently attenuated viral proliferation. These results established that PRRSV exploited the ROS/HIF-1α axis to reprogram host glucose metabolism through PPP potentiation, creating a biosynthetic environment conducive to viral propagation.

## IMPORTANCE

PRRSV remains one of the most economically devastating pathogens in swine production, yet the mechanisms by which it hijacks host metabolism to fuel replication are poorly understood. This study uncovers a novel ROS/HIF-1α/G6PD axis as a central driver of PRRSV-induced metabolic reprogramming, which provides critical mechanistic insights into how PRRSV manipulates host metabolism to create a proviral environment, bridging the gap between viral pathogenesis and host metabolic adaptation. These findings not only expand our understanding of PRRSV-host interactions but also identify the ROS-HIF-1α-G6PD signaling axis as a promising therapeutic target for disrupting viral metabolic dependency, offering a novel strategy to combat PRRSV and related RNA viruses.

## Introduction

Porcine reproductive and respiratory syndrome (PRRS) is a highly contagious disease caused by the porcine reproductive and respiratory syndrome virus (PRRSV) [1]. Since its initial outbreak in the late 1980s, the disease has spread globally and has inflicted substantial economic losses on the swine industry. PRRSV establishes persistent infection by suppressing both innate and adaptive immune responses through multiple mechanisms, leading to reproductive failure in sows and severe respiratory illness in piglets [2]. Currently, vaccination remains the primary strategy for controlling PRRS. However, the available vaccines exhibit limited cross-protection and poor immunological durability, making them insufficient to effectively contain the ongoing spread of the virus. Therefore, there is an urgent need to develop novel preventive and therapeutic approaches that are safer, more effective, and capable of providing broad-spectrum protection.

Viruses rely on host cells and their fundamental metabolic processes to complete essential steps of their life cycle, including infection, replication, and release. Upon infection, viruses hijack host metabolic pathways to acquire energy and biosynthetic intermediates necessary for their propagation. Consequently, viral replication efficiency is closely regulated by host metabolic activity. To create a favorable intracellular environment for replication, many viruses actively reprogram host carbohydrate metabolism by modulating key metabolic pathways. Under certain stimulatory conditions, cells shift their metabolic preference from glucose metabolism to mitochondrial fatty acid (FA) oxidation. Fatty acids released from lipid droplets via lipolysis or lipophagy are internalized into mitochondria, where they serve as substrates for β-oxidation and the citrate cycle. Throughout this process, lipid droplets communicate and coordinate their activities through membrane contact site-mediated mechanisms [3]. HIF-1α is a transcription factor that plays a central regulatory role in the cellular response to hypoxic stress. Moreover, HIF-1 can be activated not only by oxygen deprivation but also in response to pathogen-associated molecular patterns (PAMPs). This metabolic reprogramming driven by HIF-1 activation is known as the Warburg effect or aerobic glycolysis. Newcastle disease virus (NDV) infection leads to the loss of NAD^+^-dependent deacetylase SIRT3 through mitophagy. The absence of SIRT3 increases glycolytic metabolism by regulating the stability and activity of HIF1α [4]. hepatitis C virus (HCV) enhances glycolysis, the pentose phosphate pathway (PPP), and the tricarboxylic acid cycle to support the biosynthetic demands of replication [5]. Feline calicivirus significantly increases PPP flux in infected CRFK cells, and pharmacological inhibition of PPP effectively suppresses viral replication [6] These findings suggest that viruses can actively reshape host cell metabolism to benefit viral replication rather than maintain cellular homeostasis [7]. Furthermore, viruses can trigger cellular stress responses while hijacking host metabolites to complete their life cycle. The endoplasmic reticulum (ER) is a crucial organelle involved in the synthesis and processing of proteins, membrane lipids, and secretory proteins. During viral replication, the accumulation of large quantities of viral proteins and replication intermediates within the cell can directly induce ER stress [8]. Additionally, when the demand for rapid processing of viral proteins exceeds the normal processing capacity of the ER, it can also lead to the onset of ER stress [9]. Metabolic reprogramming triggered by pathogenic infection not only hijacks the host’s anabolic processes to support its own proliferation but also modulates the host immune response to control infection. In macrophages, this metabolic reprogramming leads to the production of fumarate via the aspartate-argininosuccinate shunt. This metabolite enhances interferon-beta (IFN-β) production by directly succinylating mitochondrial antiviral-signaling protein (MAVS) and activating retinoic acid-inducible gene-I-like receptor (RLR) signaling pathways [10]. Therefore, identifying the key factors by which viruses regulate host metabolism is critical for elucidating virus – host interaction mechanisms and offers new insights and potential targets for the development of metabolism-based antiviral therapies.

The PPP is a cytoplasmic metabolic pathway consisting of oxidative and non-oxidative branches. The oxidative branch catalyzes the oxidation of glucose-6-phosphate (G6P), producing NADPH and ribose-5-phosphate (R5P), which serve as essential substrates for nucleotide biosynthesis. The non-oxidative branch converts PPP intermediates into fructose-6-phosphate and glyceraldehyde-3-phosphate, which can feed into glycolysis and other metabolic pathways [11]. Among these products, R5P is the direct precursor for nucleic acid synthesis and is especially critical for rapidly proliferating cells, such as cancer cells or virus-infected cells. NADPH plays a central role in lipid and macromolecule biosynthesis as well as in maintaining redox homeostasis, thus contributing to the broader regulation of cellular metabolic reprogramming.

In virus-infected host cells, PPP-derived metabolites are widely recognized as major sources for nucleotide biosynthesis. Accumulating evidence has demonstrated that various viruses, including SARS-CoV-2 [12], influenza virus [13], HCV [14], and Human immunodeficiency virus (HIV) [15], can rewire host PPP activity to fulfill the energetic and biosynthetic demands of viral replication. However, whether PRRSV possesses a similar capacity to modulate the PPP in host cells remains unclear. Moreover, it is unknown whether such metabolic reprogramming is directly involved in supporting PRRSV replication. Therefore, this study aimed to systematically investigate the impact of PRRSV infection on host PPP metabolism and explore its underlying role in the viral replication process.

In the present study, we elucidated the molecular mechanisms by which PRRSV reprogramed host PPP metabolism. Specifically, PRRSV infection triggered the accumulation of reactive oxygen species (ROS), which in turn activates the HIF-1α/G6PD signaling axis. Enhanced G6PD activity promotes PPP flux, thereby providing metabolic support necessary for viral replication and facilitating efficient propagation of PRRSV in host cells.

## Materials and methods

### Pigs

Specific-pathogen-free weaned piglets (30 days old; confirmed negative for PRRSV, pseudorabies virus, porcine circovirus, and classical swine fever virus) were housed in a pathogen-controlled facility. All procedures adhered to the Guide for the Care and Use of Laboratory Animals and ethical regulations at Henan Agricultural University. Duroc piglets weighing 7–8 kg received intranasal inoculation with PRRSV strain HN07-1 (2 × 10^5^ TCID_5__0_/piglet). At 30 days post-infection, animals were humanely euthanized via intravenous administration of pentobarbital sodium (150 mg/kg) following guidelines recommended by the Chinese Association for Laboratory Animal Sciences, and tissue samples were promptly collected for downstream analysis.

### Cells and virus

MARC-145 and HEK293T cells were cultivated in Dulbecco’s Modified Eagle Medium (DMEM, Gibco, 10,566–016) supplemented with 10% fetal bovine serum (FBS, Gibco, 10099141C). Porcine alveolar macrophages (PAMs) were isolated via bronchoalveolar lavage from 4-week-old specific-pathogen-free piglets confirmed negative for pseudorabies virus, porcine circovirus, classical swine fever virus, PRRSV-1, and PRRSV-2. Isolated PAMs were maintained in RPMI 1640 medium (Gibco, 61,870,036) containing 100 U/mL penicillin, 100 μg/mL streptomycin sulfate (Sangon, B540732), and 10% FBS (Gibco, 10099141C), All cell types were grown as monolayers at 37°C under 5% CO_2_.

The PRRSV strain HN07-1 (GenBank accession no: KX766378.1) was kindly provided by Professor Gai-Ping Zhang from Henan Agricultural University [16]. The virus was propagated in MARC-145 cells, and viral titers were determined using the Reed-Muench method and expressed as 7 × 10^6^ TCID_50_/mL.

### Plasmids and transfection

The p3×FLAG-CMV-10 vector (E4401, Sigma-Aldrich) was used for cloning. G6PD and HIF-1α coding sequences were amplified from MARC-145 cDNA and inserted into this vector to generate FLAG-tagged expression constructs. E. coli TOP10 competent cells (laboratory stock) were used for plasmid propagation. Transfections employed Lipofectamine 3000 (L3000015; Invitrogen) per manufacturer’s protocol.

### Chemicals and antibodies

N-acetylcysteine (NAC, IA0050) was sourced from Solarbio. Primary antibodies against G6PD (25413–1-AP), HIF-1α (20960–1-AP), and GAPDH (10494–1-AP) were obtained from Proteintech. PRRSV-N antibody was generated in-house. HRP-conjugated secondary antibodies included goat anti-rabbit IgG (A16104) and goat anti-mouse IgG (PA1-74421) from Thermo Fisher Scientific.

### Metabolome sample preparation

Homogenized samples received 20 μL methanolic 2-chloro-1-phenylalanine (0.3 mg/mL) as an internal standard, plus 1 mL methanol:water (4:1, v/v). Mixtures were transferred to 4 mL glass vials, supplemented with 200 μL trichloromethane, and pipette-mixed. Cellular disruption was achieved via ultrasonic homogenization (500 W, 3 min). Contents were moved to 1.5 mL tubes and extracted ultrasonically in ice water (20 min). Following centrifugation (16,000 ×g, 10 min, 4°C), 1 mL supernatant was evaporated at room temperature. Dried residues were reconstituted in 200 μL methanol:water (1:4, v/v), vortexed (30 s), incubated (4°C, 2 min), and recentrifuged (16,000 ×g, 15 min, 4°C). Supernatants (150 μL) were collected via glass syringes, filtered (0.22 μm), and transferred to LC vials stored at −80°C until LC-MS analysis.

### LC-MS analysis

Analysis utilized an ACQUITY UPLC I Class system coupled to a VION IMS QTOF mass spectrometer (Waters) in ESI^+^ and ESI^−^ modes. Chromatographic separation employed an ACQUITY UPLC HSS T3 column (100 mm × 2.1 mm, 1.8 μm) under reversed-phase conditions. Ion source voltage was set to 3.5 kV (ESI^+^) or − 3 kV (ESI^−^). MS data were acquired in MSE mode (TOF range: 125–1000 Da). Mass calibration occurred after every ten samples. Quality control samples were analyzed every ten injections to ensure system stability.

### RNA interference

shRNA oligonucleotides were cloned into pLKO.1 vector (#10878, Addgene). HEK293T cells (4 × 10^6^) seeded in 10-cm dishes were co-transfected after 24 h with pLKO.1-shRNA (1 μg), pMD2.G (0.25 μg), and psPAX2 (0.75 μg). Lentiviral supernatants harvested at 48 h post-transfection infected target cells. Selection used 15 μg/mL puromycin for 7 days. shRNA sequences are listed in Table S1.

### NADPH assay

Intracellular NADPH levels were determined using a NADP^+^/NADPH Quantification Kit (S0179, Beyotime) according to the manufacturer’s instructions. The results were normalized to total protein concentration.

### EdU incorporation assay

Cells were incubated with 50 μM EdU (2 h), fixed in 4% paraformaldehyde (30 min), blocked with glycine, and permeabilized with 0.5% Triton X-100. After PBS washes, cells were treated with 1× Apollo reaction cocktail (30 min, dark), and nuclei were counterstained with DAPI (10 min). Imaging was performed using an inverted fluorescence microscope.

### Immunoblotting analysis

Cell lysis used ice-cold RIPA buffer (P0013B, Beyotime) containing protease inhibitors (HY-K0010, HY-K0022; MedChemExpress). Lysates/supernatants mixed with SDS loading buffer were denatured (98°C, 10 min). Proteins were separated via SDS-PAGE and transferred to PVDF membranes (ISEQ00010, Millipore). Membranes blocked with 5% nonfat milk (A600669, Sangon; 2 h, RT) were incubated with primary antibodies (4°C, overnight), then HRP-conjugated secondary antibodies (2 h). Bands were visualized using Luminate Crescendo substrate (WBLUR0500, Millipore) on a GE AI600 system

### qRT-PCR analysis

Total RNA extraction employed TRIzol (9108, TaKaRa), followed by cDNA synthesis with PrimeScript RT kit (RR047A, TaKaRa). qPCR used SYBR Premix Ex Taq (RR820A, TaKaRa) with ACTB as reference. Relative expression was calculated via 2^−^ ΔΔCt method. Triplicate reactions included melting curve analysis for specificity. Primers are provided in Table S1.

### MitoSOTM determination

Mitochondrial superoxide was detected using MitoSOXTM Red reagent (S0061S, Beyotime) per manufacturer’s instructions. Fluorescence images were captured with a FITC filter-equipped microscope, and intensity was quantified using ImageJ (1.8.0).

### Cell viability assay

Viability was assessed via CCK-8 assay (GK3607, DingGuo, Beijing) following the supplier’s guidelines.

### Statistical analysis

Data were processed using GraphPad Prism 8 (GraphPad Software, USA). Group comparisons employed two-tailed unpaired Student’s t-test (P vs. control). Significance was defined as *p* < 0.05. Data represent mean ± SD from three independent experiments.

## Results

### PRRSV infection activates ppp

**Figure 1.**
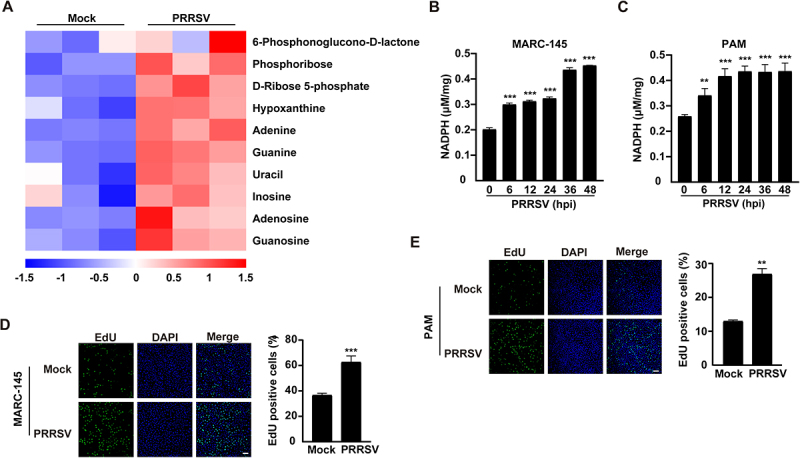
PRRSV infection activates PPP. (A) Heatmap analysis of differential metabolites involved in PPP in PAMs infected with PRRSV (MOI = 1) for 24 h compared with mock-infected controls. The color gradient from blue to red indicates the expression abundance of metabolites from low to high, with red representing higher expression levels of differential metabolites. The x-axis represents different sample groups, and the y-axis denotes the selected differential metabolites. (B and C) Quantification of intracellular NADPH levels in MARC-145 cells (B) and PAMs (C) at indicated time points (0–48 h) post PRRSV infection (MOI = 1). **p < 0.01, ***p < 0.001. (D and E) DNA synthesis analysis in MARC-145 cells (D) and PAMs (E) at 36 h post PRRSV infection (MOI = 1) using EdU incorporation assay. **p < 0.01, ***p < 0.001.

Based on our previous metabolomic data, PRRSV infection exerts a significant impact on the PPP [17]. To further validate this observation, we performed hierarchical clustering analysis of PPP-related differential metabolites. As shown in Figure 1(A), the abundance of metabolites in the PPP and its downstream nucleotide biosynthesis pathways was markedly increased in PRRSV-infected PAMs. We next evaluated whether PRRSV infection activates PPP. The results demonstrated that, compared to the control group, PRRSV infection significantly elevated the levels of key PPP metabolites NADPH, in both MARC-145 and PAMs (Figure 1(B,C)). Given that PPP-derived metabolites serve as precursors for DNA synthesis, we further assessed DNA synthesis activity. Notably, PRRSV infection markedly promoted DNA synthesis in host cells (Figure 1(D,E)). Collectively, these results indicate that PRRSV infection reprograms host cell metabolism by enhancing PPP activity to facilitate biosynthetic processes necessary for viral replication.

### PRRSV infection upregulates G6PD expression

**Figure 2.**
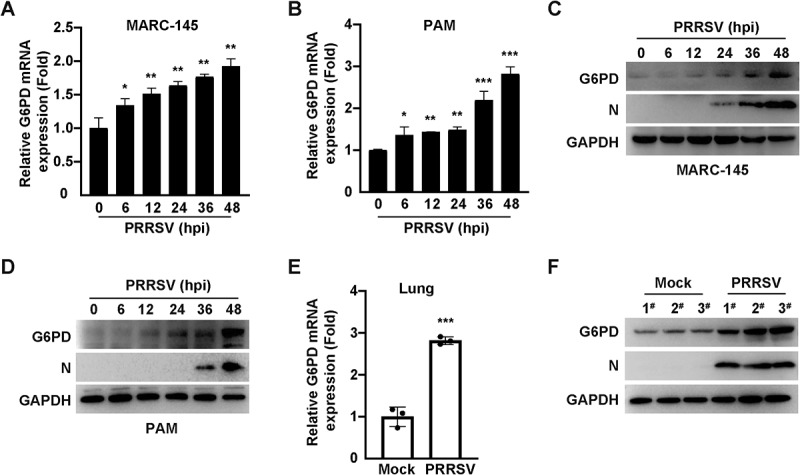
PRRSV infection upregulates G6PD expression. (A and B) Relative mRNA expression levels of G6PD in MARC-145 cells (A) and PAMs (B) infected with PRRSV (MOI = 1) for 0–48 h, as determined by qRT-PCR. *p < 0.05, **p < 0.01, ***p < 0.001. (C and D) Protein levels of G6PD and PRRSV N in MARC-145 cells (C) and PAMs (D) at 0–48 h post PRRSV infection (MOI = 1), analyzed by immunoblotting analysis. (E) qRT-PCR analysis of G6PD mRNA expression in lung tissues from piglets intranasally infected with PRRSV (2 × 10^5^ TCID_50_/pig) or mock-infected for 30 days. Three piglets per group. ***p < 0.001. (F) Immunoblotting analysis of G6PD and PRRSV N protein levels in lung tissues of piglets treated as described in (E). Three piglets per group.

G6PD, the first and rate-limiting enzyme of the PPP, catalyzes the conversion of G6P to R5P. Its enzymatic activity directly influences PPP flux and overall cellular metabolic status. To investigate the role of G6PD during PRRSV infection, we established PRRSV infection models in MARC-145 cells and PAMs cells, and examined G6PD mRNA and protein expression levels using qRT-PCR and immunoblotting analysis, respectively. The results showed that PRRSV infection significantly increased G6PD mRNA levels in both cell types (Figure 2(A,B)), accompanied by a marked upregulation of G6PD protein expression (Figure 2(C,D)). To further determine whether PRRSV induces G6PD expression *in vivo*, piglets were either mock-treated or intranasally inoculated with PRRSV, and lung tissues were collected for analysis 30 days post-infection (Figure 2(E)). Compared with the control group, the PRRSV-infected piglets exhibited significantly elevated G6PD mRNA expression in lung tissues (Figure 2(F)), along with increased protein levels (Figure 2(G)). Together, these findings demonstrate that PRRSV markedly induces G6PD expression both *in vitro* and *in vivo*, suggesting that G6PD may play a critical role in virus-induced metabolic reprogramming.

### G6PD facilitates PRRSV replication

**Figure 3.**
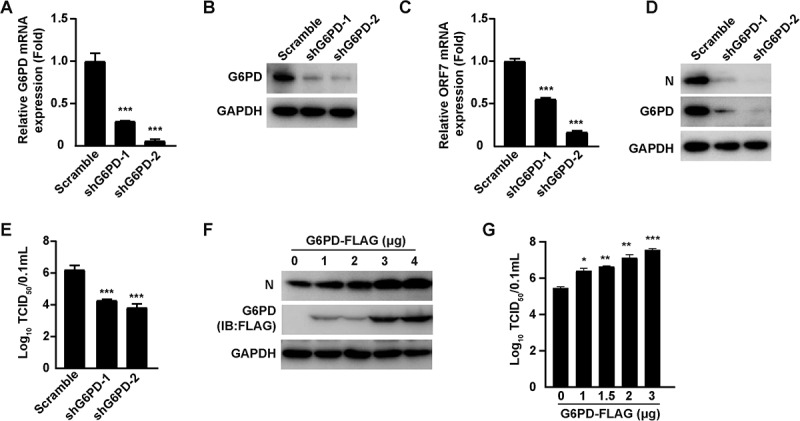
G6PD facilitates PRRSV replication. (A and B) qRT-PCR (A) and immunoblotting (B) analyses of G6PD mRNA and protein expression levels, respectively, in MARC-145 cells transduced with scramble control, shG6PD-1, or shG6PD-2 lentiviral vectors. ***p < 0.001. (C) Relative mRNA expression levels of PRRSV N in scramble, shG6PD-1, and shG6PD-2 MARC-145 cells infected with PRRSV (MOI = 1) for 36 h, analyzed by qRT-PCR. ***p < 0.001. (D) Immunoblotting analysis of PRRSV N and G6PD protein levels in scramble, shG6PD-1, and shG6PD-2 MARC-145 cells infected with PRRSV (MOI = 1) for 48 h. (E) Virus titers in the supernatants of scramble, shG6PD-1, and shG6PD-2 MARC-145 cells infected with PRRSV (MOI = 1) for 48 h, determined by the TCID_50_ assay. ***p < 0.001. (F) MARC-145 cells were transfected with increasing amounts (0–4 μg) of G6PD-FLAG plasmid for 12 h, followed by PRRSV infection (MOI= 1) for 36 h. Protein levels of PRRSV N and G6PD-FLAG were analyzed by immunoblotting analysis. (G) MARC-145 cells were transfected with increasing amounts (0–3 μg) of G6PD-FLAG plasmid for 12 h, followed by PRRSV infection (MOI = 1) for 48 h. Virus titers were determined by the TCID_50_ assay. *p < 0.05, **p < 0.01, ***p < 0.001.

To elucidate the role of G6PD in PRRSV infection, we employed RNA interference using short-hairpin RNAs (shRNAs) targeting G6PD. qRT-PCR and immunoblotting analysis confirmed that G6PD expression was markedly downregulated in shG6PD-1 and shG6PD-2 cells, as compared to the scramble control (Figure 3(A,B)). Subsequently, the control (Scramble) and G6PD-knockdown cells were infected with PRRSV, and the expression levels of viral ORF7 mRNA and N protein were assessed. The results showed that G6PD knockdown significantly reduced the expression of PRRSV ORF7 mRNA (Figure 3(C)), and N protein levels were markedly lower in both shG6PD-1 and shG6PD-2 groups compared to the scramble control (Figure 3(D)). Consistently, viral titer analysis further demonstrated that PRRSV replication was significantly impaired in G6PD-knockdown cells (Figure 3(E)). To further validate the positive regulatory role of G6PD in PRRSV proliferation, MARC-145 cells were transfected with an empty vector or increasing amounts of a FLAG-tagged G6PD expression plasmid, followed by PRRSV infection. Immunoblotting analysis revealed that PRRSV N protein levels increased in a G6PD dose-dependent manner (Figure 3(F)). Moreover, viral titers were elevated upon ectopic expression of G6PD-FLAG (Figure 3(G)). Together, these data clearly demonstrate that G6PD facilitates PRRSV proliferation.

### PRRSV infection induces HIF-1α activation

**Figure 4.**
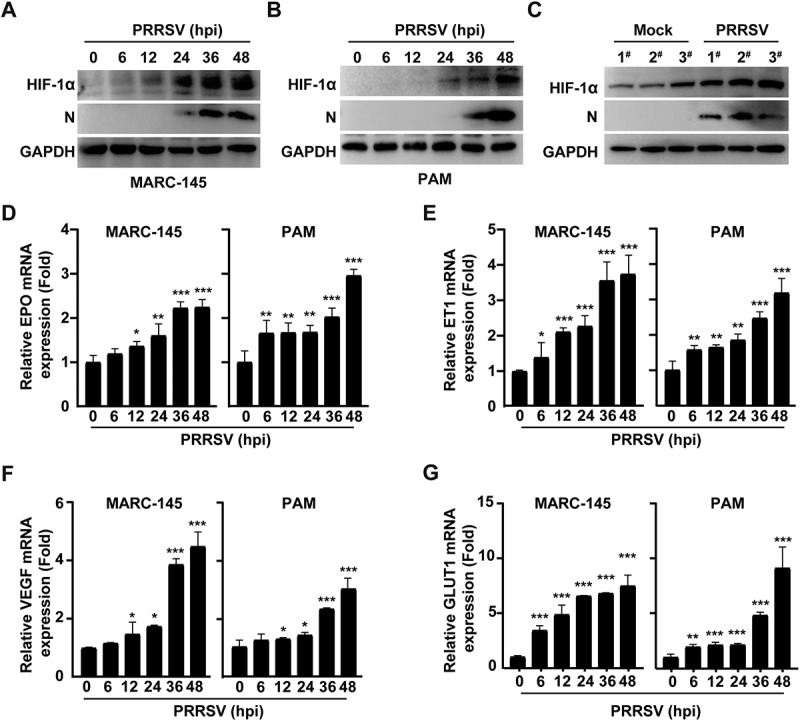
PRRSV infection induces HIF-1α activation. (A and B) Immunoblotting analysis of HIF-1α and PRRSV N protein levels in MARC-145 (A) and PAMs (B) cells infected with PRRSV (MOI = 1) for 0–48 h. (C) Piglets were mock-infected or intranasally infected with PRRSV at a dose of 2 × 10^5^ TCID_50_/head and sacrificed at 30 days post-infection. HIF-1α and PRRSV N protein levels in lung tissues were detected by immunoblotting analysis. (D-G) qRT-PCR analysis of the mRNA expression levels of HIF-1α downstream target genes, including epo (D), ET1 (E), VEGF (F) and GLUT1 (G), in MARC-145 and PAMs cells infected with PRRSV (MOI = 1) for 0–48 h. *p < 0.05, **p < 0.01, ***p < 0.001.

Previous studies have shown that hypoxic conditions can induce the expression of G6PD, with hypoxia-inducible factor 1-alpha (HIF-1α) being a key transcriptional regulator in cellular responses to hypoxia [18]. To investigate whether PRRSV induces G6PD expression via HIF-1α, we examined the protein levels of HIF-1α in PRRSV-infected MARC-145 and PAMs. Immunoblotting analysis revealed a significant upregulation of HIF-1α expression in both cell types following PRRSV infection (Figure 4(A,B)). To determine whether a similar effect occurs in vivo, we analyzed lung tissues from piglets 30 days after intranasal or mock PRRSV infection. The results showed that HIF-1α protein levels were markedly increased in lung tissues from the PRRSV-infected group compared to the mock-infected group (Figure 4(C)). Furthermore, to confirm HIF-1α activation by PRRSV, we assessed the mRNA levels of known HIF-1α downstream targets, including erythropoietin (EPO), endothelin-1 (ET1), vascular endothelial growth factor (VEGF) and glucose transporter 1 (GLUT1). qRT-PCR analysis showed that the transcription of these genes was significantly upregulated in both MARC-145 and PAMs upon PRRSV infection (Figure 4(D–G)). These findings collectively indicate that PRRSV infection induces HIF-1α expression and activates its downstream signaling pathways.

### HIF-1α promotes PRRSV replication

**Figure 5.**
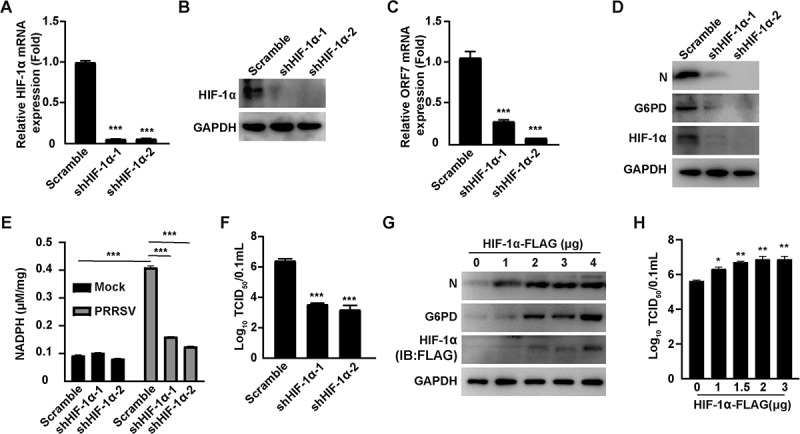
HIF-1α promotes PRRSV replication. (A and B) qRT-PCR (A) and immunoblotting (B) analysis of HIF-1α mRNA and protein expression levels in MARC-145 cells transfected with scramble, shHIF-1α-1, and shHIF-1α-2. ***p < 0.001. (C) MARC-145 cells transfected with scramble, shHIF-1α-1, shHIF-1α-2, and PRRSV (MOI = 1) were cultured for 36 h. PRRSV N mRNA expression levels were analyzed by qRT-PCR. ***p < 0.001. (D) MARC-145 cells transfected with scramble, shHIF-1α-1, shHIF-1α-2, and PRRSV (MOI = 1) were cultured for 48 h. PRRSV N and HIF-1α protein levels were detected by immunoblotting analysis. (E) Quantification of intracellular NADPH levels in scramble, shHIF-1α-1, and shHIF-1α-2 MARC-145 cells either mock-infected or infected with PRRSV infection (MOI = 1) for 48 h. ***p < 0.001. (F) MARC-145 cells transfected with scramble, shHIF-1α-1, shHIF-1α-2, and PRRSV (MOI= 1) were cultured for 48 h. Virus titers were measured by TCID_50_ method. ***p < 0.001. (G) MARC-145 cells were transfected with increasing concentrations of HIF-1α-FLAG plasmid (0–4 μg) for 12 h, followed by PRRSV infection (MOI = 1) for 36 h. Immunoblotting analysis was performed to detect PRRSV N and HIF-1α-FLAG protein levels. (H) MARC-145 cells were transfected with increasing concentrations of HIF-1α-FLAG plasmid (0–3 μg) for 12 h, followed by PRRSV infection (MOI = 1) for 48 h. Virus titers were measured by TCID_50_ method. *p < 0.05, **p < 0.01.

To elucidate the role of HIF-1α in PRRSV infection, we silenced its expression using shRNA-mediated knockdown. qRT-PCR and immunoblotting analysis confirmed that both shHIF-1α-1 and shHIF-1α-2 effectively reduced HIF-1α expression in MARC-145 cells (Figure 5(A,B)). Subsequently, scramble control and HIF-1α knockdown cells were infected with PRRSV, and the expression levels of viral ORF7 mRNA and N protein were measured. HIF-1α knockdown significantly decreased ORF7 mRNA levels (Figure 5(C)) and resulted in markedly reduced N protein expression (Figure 5(D)). Notably, suppression of HIF-1α also led to a significant reduction in G6PD protein expression, with both shHIF-1α-1 and shHIF-1α-2 exhibiting similar trends compared to the scramble control. In addition, we measured the level of NADPH, a key metabolite of the PPP. PRRSV infection significantly increased NADPH production, whereas knockdown of HIF-1α markedly attenuated this elevation (Figure 5(E)). Consistently, viral titer assays revealed that PRRSV production was significantly impaired in HIF-1α-silenced cells (Figure 5(F)). To further validate the positive regulatory role of HIF-1αin PRRSV proliferation, MARC-145 cells were transfected with an empty vector or increasing amounts of a FLAG-tagged HIF-1α expression plasmid, followed by PRRSV infection. Immunoblotting analysis demonstrated that N protein levels increased in a dose-dependent manner with elevated HIF-1α expression (Figure 5(G)). Intriguingly, G6PD protein levels were also upregulated in parallel, suggesting that HIF-1α may enhance PRRSV replication by modulating G6PD expression. Viral titer analysis confirmed that ectopic expression of HIF-1α-FLAG significantly promoted PRRSV replication (Figure 5(H)). Together, these results support the conclusion that PRRSV promotes its replication by inducing HIF-1α, which in turn upregulates G6PD expression.

### PRRSV activates HIF-1α via ROS accumulation

**Figure 6.**
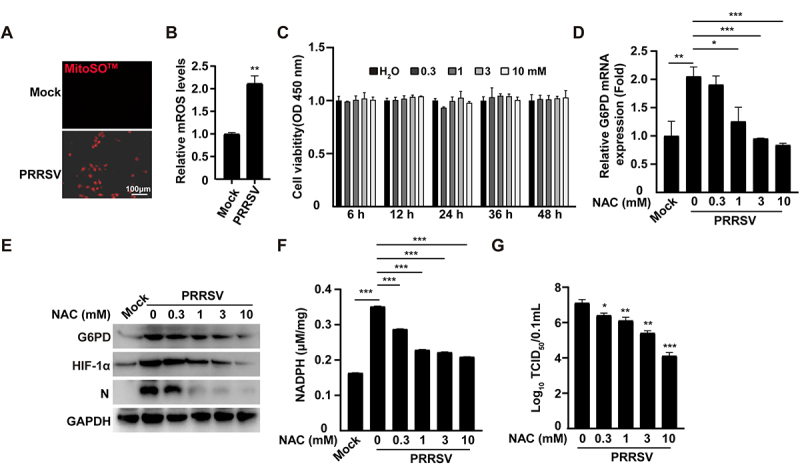
PRRSV activates HIF-1α via ROS accumulation. (A) MARC-145 cells were infected with PRRSV (MOI = 1) for 36 h and stained with MitoSO^TM^ probe for 60 minutes. Fluorescence microscopy was used to capture images. (B) Fluorescence intensity was analyzed using ImageJ software based on the images in (A). ***p < 0.001. (C) MARC-145 cells were treated with nac (0–10 mM) for the indicated times. Cell proliferation was determined using the CCK-8 assay. (D) MARC-145 cells were infected with PRRSV (MOI = 1) and treated with either vehicle or nac (0–10 mM) for 36 h. G6PD mRNA expression levels were measured by qRT-PCR. *p < 0.05, **p < 0.01, ***p < 0.001.(E) MARC-145 cells were infected with PRRSV (MOI = 1) and treated with either vehicle or nac (0–10 mM) for 36 h. G6PD and HIF-1α protein levels were detected by immunoblotting analysis. (F) MARC-145 cells were infected with PRRSV (MOI= 1) and treated with either vehicle or nac (0–10 mM) for 36 h. NADPH levels were measured using commercial kits. *p < 0.05, **p < 0.01, ***p < 0.001. (G) MARC-145 cells were infected with PRRSV (MOI = 1) and treated with either vehicle or nac (0–10 mM) for 48 h. Virus titers were measured by TCID_50_. *p < 0.05, **p < 0.01, ***p < 0.001.

Under normoxic conditions, HIF-1α is typically degraded rapidly through the ubiquitin – proteasome pathway. However, under hypoxic conditions or elevated ROS levels-such as through the accumulation of H_2_O_2_ or NO, HIF-1α degradation can be suppressed. This is mainly due to the inhibition of prolyl hydroxylase activity, leading to the stabilization of HIF-1α protein [19]. Our previous research has shown that PRRSV stimulates mitochondrial ROS to facilitate viral replication [20]. To investigate whether the PRRSV stabilizes HIF-1α through the accumulation of ROS in mitochondria, we measured the ROS levels within mitochondria using the mitochondrial superoxide kit (MitoSO^TM^). The results showed that the MitoSO^TM^ Red fluorescence intensity in PRRSV-infected cells was significantly higher than that in the uninfected control group, indicating that PRRSV infection leads to a significant accumulation of ROS in mitochondria (Figure 6(A,B)). To further explore whether PRRSV-induced ROS promotes HIF-1α activation and subsequently upregulates G6PD expression, we employed the ROS scavenger N-acetylcysteine (NAC) and assessed the resulting molecular changes. Cell viability assessed by the CCK-8 indicated that the concentrations of NAC were harmless to the cells (Figure 6(C)). qRT-PCR analysis demonstrated that PRRSV infection markedly upregulated G6PD mRNA expression. However, G6PD mRNA levels gradually decreased with increasing concentrations of NAC, and were nearly restored to baseline levels in the 10 mM NAC treatment group (Figure 6(D)). Immunoblotting analysis further confirmed that PRRSV infection elevated both G6PD and HIF-1α protein levels, which were dose-dependently inhibited by NAC treatment (Figure 6(E)). These results indicate that NAC effectively blocks ROS-mediated stabilization of HIF-1α, thereby suppressing the transcriptional and translational upregulation of G6PD. Moreover, we measured levels of the key PPP metabolites NADPH. PRRSV infection significantly enhanced the production of NADPH, while NAC treatment resulted in a dose-dependent reduction in their levels (Figure 6(F)). Consistently, viral titer assays showed that NAC treatment significantly impaired PRRSV replication (Figure 6(G)). Collectively, these findings suggest that PRRSV promotes ROS accumulation to stabilize HIF-1α expression, which in turn upregulates G6PD and activates PPP to support viral replication. The ROS scavenger NAC effectively disrupts this pathway, thereby suppressing PRRSV proliferation.

## Discussion

Following infection of host cells, viruses rely on cellular organelles, energy supply, and biosynthetic materials to efficiently replicate and produce infectious progeny. To meet these replication demands, viruses have evolved diverse strategies to reprogram host metabolic pathways, thereby supporting viral growth and proliferation. For instance, some viruses enhance glycolysis to substantially increase the production of ATP and biosynthetic precursors, thereby facilitating viral gene replication, protein synthesis, and virion assembly. Similarly, under certain pathological conditions, the organism undergoes metabolic reprogramming that shifts cellular energy metabolism from mitochondrial oxidative phosphorylation toward a greater reliance on glycolysis [21]. The PPP is a critical metabolic branch that contributes to carbon rearrangement, the biosynthesis of nucleotides and other macromolecules, and maintenance of redox homeostasis. PPP consists of two major branches: the non-oxidative branch, which interconnects with glycolysis and other metabolic pathways, and the oxidative branch, which primarily generates R5P and NADPH. R5P is an essential precursor for nucleotide biosynthesis, playing a key role during the rapid synthesis of viral nucleic acids. NADPH supports the synthesis of lipids and other macromolecules, and promotes GSH production, thereby enhancing cellular resistance to oxidative stress and preventing apoptosis-contributing to the overall metabolic reprogramming of the host. An increasing body of evidence indicates that viruses promote their replication by activating the PPP. For instance, in influenza virus-infected cells, the key PPP enzymes G6PD and 6-phosphogluconate dehydrogenase are significantly upregulated, accompanied by enhanced NADPH and nucleotide biosynthesis, which facilitates viral replication [13]. Similarly, elevated G6PD expression and NADPH levels have been observed in models of latent HCV infection [14], and in HIV-latently infected cells, increased G6PD expression and NADPH production are noted, alongside partial redirection of glycolytic intermediates toward the PPP [15]. However, whether PRRSV modulates host PPP to support its replication had not been previously elucidated. In this study, we reveal for the first time the molecular mechanism by which PRRSV reprograms host PPP to facilitate its replication. Metabolomic analysis demonstrated that PRRSV infection significantly elevated the levels of PPP-related metabolites. Mechanistically, PRRSV infection induced intracellular ROS accumulation, which stabilized and activated HIF-1α, thereby promoting G6PD expression and enhancing PPP flux to meet the nucleotide and reductive demands of viral replication.

G6PD is the rate-limiting enzyme of the PPP, catalyzing the conversion of G6P into 6-phosphogluconolactone while generating NADPH. As a key regulatory enzyme, G6PD controls the oxidative branch flux of PPP and plays an essential role in maintaining redox balance and providing nucleotide precursors such as R5P. Recent studies have demonstrated that G6PD plays a pivotal role in various viral infections, wherein viruses upregulate G6PD to reprogram host metabolism for energy and precursor synthesis. For example, Zika virus infection induces metabolic reprogramming and reroutes part of the glycolytic carbon flux into PPP, possibly by upregulating G6PD and other key enzymes [22]. Similarly, HPV16 E6 promotes cervical cancer cell proliferation by enhancing G6PD activity to activate PPP [23]. However, it is noteworthy that some viruses exhibit the opposite regulatory effect. Influenza virus infection was reported to suppress G6PD expression and activity, leading to ROS accumulation, which paradoxically benefits viral replication [24]. In this study, we systematically investigated PRRSV-induced regulation of G6PD expression and found that PRRSV significantly upregulated G6PD levels both in vitro and in vivo. Furthermore, knockdown of G6PD significantly inhibited viral replication, indicating a critical role of G6PD in PRRSV propagation. These findings suggest that PRRSV enhances PPP activity via G6PD upregulation, which not only generates NADPH for lipid and macromolecule synthesis, but also provides nucleotide precursors, thereby supporting viral replication.

Previous studies have shown that hypoxic conditions can induce G6PD expression [25], with HIF-1α being a central transcription factor in response to hypoxic stress. Under normoxia, HIF-1α is hydroxylated by PHDs, ubiquitinated, and subsequently degraded by the proteasome. Under hypoxia or oxidative stress, PHD activity is inhibited, allowing HIF-1α to escape degradation, accumulate in the nucleus, and dimerize with HIF-1β. The HIF-1α/β complex binds to hypoxia response elements in target gene promoters to initiate transcription. HIF-1α plays key roles in cellular metabolism, angiogenesis, cell cycle progression, inflammation, immune regulation, and tumor development and metastasis [26,27]. Notably, HIF-1α also promotes viral replication by modulating host metabolism. The function of HIF-1α varies depending on the virus. For example, in HIV-1-infected CD4^+^ T cells, viral cytoplasmic dsDNA stabilizes HIF-1α and enhances viral replication [28]. White spot syndrome virus activates HIF-1α to promote fatty acid synthase expression, increasing long-chain fatty acid synthesis to support virion assembly [29]. In H1N1-infected alveolar epithelial cells, although HIF-1α mRNA levels remain unchanged, and its hydroxylation and ubiquitination are unaffected, the virus inhibits proteasome activity to promote HIF-1α accumulation [30]. Conversely, in vesicular stomatitis virus infection, HIF-1α exhibits antiviral activity by upregulating type I interferons (e.g. IFN-β), enhancing host antiviral defense and restricting viral replication [31]. In studies of SARS-CoV-2, which shares structural similarities with PRRSV, it was found that hypoxia and the HIF prolyl hydroxylase inhibitor Roxadustat reduce ACE2 expression and inhibit SARS-CoV-2 entry and replication in lung epithelial cells via an HIF-1α-dependent pathway [32]. In this study, we found that PRRSV infection induces HIF-1α expression, thereby transcriptionally activating its downstream target G6PD and enhancing PPP flux to provide a metabolic advantage for viral replication. We also observed that suppression of HIF-1α expression markedly induced NADPH collapse. Cytosolic NADP is recycled to NADPH by the oxidative pentose-phosphate pathway (oxPPP), malic enzyme 1(ME1) and isocitrate dehydrogenase 1(IDH1). Whether this collapse phenomenon is modulated by additional regulatory pathways warrants further investigation. Our findings reveal a novel mechanism by which HIF-1α facilitates PRRSV replication, offering new insights into virus-host metabolic interactions.

Moreover, ROS play a crucial role in the stabilization and activation of HIF-1α [19]. In eukaryotic cells, mitochondria are the primary source of ROS. Environmental stress or pathogen infection can impair mitochondrial function and lead to excessive ROS generation. For instance, the SARS-CoV-2 ORF3a protein disrupts mitochondrial structure to induce mitochondrial ROS release, thereby elevating HIF-1α levels and promoting viral replication and host inflammation [33]. Similar interactions have been reported for HPV [34] and HCV [35], both of which induce ROS to enhance HIF-1α expression and support viral replication. Our previous studies demonstrated that PRRSV infection is associated with mitochondrial dysfunction and significantly increases ROS levels [20]. In this study, we further found that PRRSV-induced HIF-1α expression is dependent on ROS accumulation. Notably, ROS inhibition significantly suppressed HIF-1α expression and G6PD upregulation, leading to reduced viral progeny production. These findings indicate that ROS promotes HIF-1α stabilization and activation, playing a key role in PRRSV replication.

In summary, this study is the first to elucidate the mechanism by which PRRSV activates PPP to promote its own replication. Mechanistically, PRRSV induces intracellular ROS accumulation, which stabilizes and activates HIF-1α. HIF-1α, in turn, transcriptionally upregulates G6PD, enhancing PPP activity. This metabolic reprogramming provides sufficient reducing power (NADPH) and nucleotide precursors (e.g. R5P), creating a favorable intracellular environment for efficient viral proliferation. These findings not only expand our understanding of PRRSV – host interactions, but also highlight HIF-1α and G6PD as potential therapeutic targets for antiviral intervention, with important theoretical and practical implications.

## Data Availability

The datas that support the findings of this study are openlyavailable in “Mendeley Data” at https://data.mendeley.com/datasets/75py8kt44x/2 [36], doi:10.17632/75py8kt44x.2

## Arrive guidelines compliance statement

We adhered to the ARRIVE guidelines and have uploadedthe completed checklist at https://data.mendeley.com/datasets/75py8kt44x/2, doi:10.17632/75py8kt44x.2
